# Neuroprotective effect of ripasudil on retinal ganglion cells via an antioxidative mechanism

**DOI:** 10.1007/s10384-025-01243-x

**Published:** 2025-07-02

**Authors:** Reiko Yamagishi-Kimura, Megumi Honjo, Makoto Aihara

**Affiliations:** Department of Ophthalmology, Graduate School of Medicine, University of Tokyo, 7-3-1 Hongo Bunkyo-ku, Tokyo, 113-8655 Japan

**Keywords:** Glaucoma, Mouse, Neuroprotection, Retinal damage, Retinal ganglion cell, Rho-kinase inhibitor

## Abstract

**Purpose:**

To evaluate the neuroprotective effect of ripasudil, a rho-kinase inhibitor that is a commercially available glaucoma medication that lowers intraocular pressure. We explored the effects of ripasudil on retinal damage via oxidative stress (OS) in primary rat retinal ganglion cell (RGC) cultures and NMDA-induced retinal damage in mice.

**Study design:**

Experimental investigation

**Methods:**

Primary rat RGCs were isolated via a 2-step immunopanning method and cultured under normal cultivation conditions for 72 h and for a further 24 h in antioxidant-free medium for OS. We measured the number of living RGCs by use of calcein-AM and calpain activity via calpain immunoreactivity assays. Furthermore, we evaluated the effects of ripasudil via RGC counting in retinal flat-mounts from Thy1-CFP mice, retinal thickness via optical coherence tomography, and reduced glutathione levels via GSSG/GSH assays in NMDA-induced retinal damage.

**Results:**

The living RGC counts of normal, OS, 0.1, 1, 10, and 100 uM ripasudil under OS were 236.0 ± 21.6, 155.0 ± 13.2, 155.9 ± 17.1, 158.9 ± 12.0, 184.8 ± 26.9, and 201.1 ± 24.8 cells, respectively. 10 or 100 uM ripasudil significantly inhibited the OS-induced RGC reduction (*P* < 0.05 or 0.01). Furthermore, the enhanced calpain activity induced by OS was suppressed by 100 uM ripasudil (*P* < 0.05). In an in vivo study, the RGC counts in the NMDA-treated group were lower than those of the non-NMDA-treated group. NMDA-induced RGC loss was significantly suppressed by ripasudil (*P* < 0.01). Retinal thinning after 3 weeks of NMDA injection was also inhibited by ripasudil (*P* < 0.01 or 0.05). Furthermore, NMDA increased the glutathione level, whereas ripasudil suppressed it (*P* < 0.05).

**Conclusions:**

Ripasudil may have neuroprotective effects via an antioxidative mechanism, which could be useful as an intraocular pressure-independent additive.

## Introduction

Glaucoma is a characteristic ocular disease caused by optic neuropathy and is one of the main causes of visual field defects [1]. On a global basis, in 2020, approximately 3.16 million people were blind; glaucoma accounted for 8.39% of all blind people and for visual impairment in 4.14 million people [2]. Although the pathogenesis of glaucoma remains unclear, the consensus is that progressive degeneration of retinal ganglion cells (RGCs) is a key cellular event that leads to characteristic glaucomatous optic disc changes with associated visual field loss [3]. Current treatment strategies for lowering intraocular pressure (IOP) are based on robust evidence from animal experiments showing the close relationship between IOP level and RCG death [4] and major randomized controlled trials showing the protective effect of lowering IOP on visual field progression in glaucoma eyes [5]. However, in Asia, the majority of patients are diagnosed with glaucoma classified as normal tension glaucoma (NTG), and recent epidemiologic studies have revealed that visual field loss cannot be prevented by lowering IOP alone [6, 7]. In addition, a number of studies have suggested that dysregulation of blood flow (particularly in NTG patients) plays an important role in the development of glaucoma [8–12]. Close relationships between retinal nerve fiber layer thickness and 8-OHdG (an oxidative stress marker) levels [13], increased serum lipids and oxidative stress in NTG [14], increased oxidative stress level in glaucomatous marmosets [15], and increased systemic oxidative stress levels and reduced ocular blood flow in NTG patients [13, 16] have also been reported. These findings suggest that IOP-independent neuroprotection of RGCs may be a new approach for glaucoma treatment.

Ripasudil is the world’s first rho-kinase inhibitor eye drop (Glanatec ophthalmic solution 0.4%; Kowa) that reduces IOP in mice, rabbits, monkeys, and humans. The IOP-lowering mechanism increases conventional aqueous outflow via the trabecular meshwork modulating the trabecular meshwork cell cytoskeleton, adjusting the extracellular matrix composition and Schlemm canal by increasing SC permeability [17–19].

Furthermore, a previous study revealed that rho-associated coiled-coil kinase (ROCK) inhibitors have various pharmacologic activities, such as augmentation of optic nerve head blood flow [20]; NO-independent increases in optic nerve head blood flow and vasodilation of ciliary arteries in rabbits [21]; antiapoptotic effects [22]; antioxidant effects [23]; and anti-inflammatory effects on microglia [24]. Thus, ROCK inhibitors not only have a strong IOP-lowering effect but also may be beneficial for the glaucomatous pathogenesis of retinal damage by improving blood flow through vasodilation and reducing retinal cell death through multiple mechanisms. However, the details of the mechanism by which ripasudil affects RGC death and retinal damage remain unclear. In view of the above findings, the present study was designed to investigate in detail the effects of the ROCK inhibitor ripasudil on damage to primary rat RGC cells and in mice, with a focus on oxidative stress both in vivo and in vitro.

## Materials and methods

### Materials

N-methyl-D-aspartic acid (NMDA), poly-L-lysine, bovine serum albumin (BSA), L-glutamine, human recombinant brain-derived neurotrophic factor (BDNF), and rat recombinant ciliary neurotrophic factor (CNTF) were obtained from Sigma‒Aldrich. Forskolin was purchased from Fujifilm. Laminin was obtained from Thermo Fisher Scientific. The papain dissociation system was obtained from Worthington Biochemical; mouse anti-rat SIRP (CD172a) monoclonal antibodies (MAB 1407P) and mouse anti-rat Thy1.1 monoclonal antibodies (MAB 1406) were obtained from Merck Millipore. A live/dead viability cytotoxicity kit was obtained from Molecular Probes. Neurobasal medium, B27 supplement, and B27 supplement minus antioxidants (AO-) were obtained from Gibco. Unless otherwise stated, the B27 supplement contains antioxidants. Ripasudil was obtained from Kowa.

### Purification and culture of rat RGCs

RGCs and retinal glial cells were purified via a 2-step immunopanning procedure, as described previously [25, 26]. Briefly, 5-day-old Wistar rats were euthanized, and the eyes, enucleated. The collected retinas were dissociated into a cell suspension via a papain dissociation system. The cell suspension was incubated for 30 min in a tube coated with an antimacrophage monoclonal antibody at room temperature. The cells that did not adhere to the antimacrophage antibody were transferred and incubated for 60 min in a tube coated with the anti-Thy1.1 monoclonal antibody at room temperature. The cells that adhered to the anti-Thy1.1 antibody were cultured as RGCs in serum-free neurobasal medium supplemented with 2% B27, 40 ng/mL BDNF, 40 ng/mL CNTF, 1 mM L-glutamine, 10 mM forskolin, 100 U/mL penicillin, and 100 mg/mL streptomycin. RGCs were cultured on plates coated with 50 mg/mL poly-L-lysine, and 1 mg/mL laminin at 2 × 10^5^ cells/well in 24-well plates. After 72 h, the RGCs were used for the experiments.

### Oxidative stress and measurement

After 72 hours of cultivation under normal conditions, the RGCs were pretreated with ripasudil for 1 h and subsequently exposed to oxidative stress in B27 medium without antioxidants (AO-) for 24 h. Control coverslips were moved to freshly prepared B27-supplemented neurobasal medium containing potent antioxidants (reduced glutathione, vitamin E, vitamin E acetate, catalase, and superoxide dismutase), and coverslips for oxidative treatment were transferred to neurobasal medium containing B27 without these 5 antioxidants (AO-), a condition conducive to oxidative stress. Each assay was repeated 6 times with or without ripasudil. After oxidative stress, the number of RGCs was determined via a cell viability assay. For the counting of RGCs, the number of viable cells was measured at 8 locations on the abyssal side of the 24-well plate with the added drug masked. RGC viability under each condition was evaluated in comparison with that under normal conditions. Furthermore, calpain activation by oxidative stress with or without ripasudil was assessed via calpain immunoreactivity assays.

### Mice

All the mice were treated in accordance with the ARVO Statement for the Use of Animals in Ophthalmic and Vision Research and the dictates of our local animal use committee at the University of Tokyo. The protocol for the animal experiments used in this study was approved by the University of Tokyo’s animal ethics committee. All the experiments were performed in accordance with the relevant named guidelines, regulations, and ARRIVE guidelines. Male Thy-1 CFP mice were obtained from Kyoto University. The mice were bred and housed in clear cages covered loosely with air filters. The cages contained white-chip bedding. The temperature was maintained at 21 °C with a 12-hour light (6:00 am—6:00 pm) and a 12-hour dark cycle. After purchase, all the mice had access to food and water ad libitum in a conventional animal room in our laboratory for at least 1 week before the scheduled experimental date. For all the experiments, 8–10-week-old male mice (with body weights ranging from 18 to 24 g) were used.

### NMDA-induced retinal damage in mice

Retinal damage in mice was induced via intravitreal injection of NMDA, as previously reported [27]. Briefly, the mice were anesthetized via intraperitoneal administration of 80 mg/kg ketamine HCl (Ketalar; Daiichi Sankyo) and 10 mg/kg xylazine (Celectal; Bayer). The pupils were dilated with a solution of 0.5% tropicamide phenylephrine hydrochloride (Mydrin-P; Santen), and 0.4% oxybuprocaine hydrochloride (Benoxil; Santen) was administered topically as an anesthetic. Retinal damage was induced by the intravitreal injection of 2 μL of 10 mM NMDA dissolved in phosphate-buffered saline (PBS; Fujifilm) via a Hamilton syringe with a 32-gauge needle. For coadministration of ripasudil and NMDA, a mixed solution of 20 mM NMDA and each concentration of ripasudil in equal amounts was prepared, and 2 μL of intravitreal injection was administered. The same volume of injected PBS was used as the vehicle control. The nontreated group served as the control. Ofloxacin ophthalmic ointment (Tarivid ophthalmic ointment 0.3%; Santen) was applied topically to the treated eye immediately after intravitreal injection. If hyphema or vitreous hemorrhage occurred, then these mice were excluded from the experiment. The mice were classified into 5 groups: nontreated group (normal), NMDA group (control), and NMDA + ripasudil groups (1, 10, or 100 µM ripasudil).

### Measurement of the RGC count

One week after intravitreal injection, the number of retinal ganglion cells (RGCs) in the retinal flat mounts was counted. The mice were sacrificed, and the eyes were enucleated and immediately fixed in 4% paraformaldehyde in 0.1 M PBS for 1 h at 4 °C. Four radial relaxing incisions were made, and the retina was prepared as a flattened whole mount on a glass slide with a coverslip. Images were obtained via a fluorescence microscope (BX50; Olympus) with a CFP filter set. The number of RGCs expressing CFP was manually counted via image-processing software (ImageJ) in 12 separate areas of 40,000 μm^2^. Areas that were 600 μm (central), 1200 μm (middle), and 1800 μm (peripheral) away from the optic disc in each of the retinal quadrants were sampled. The average density of RGCs/mm^2^ for each retinal area was obtained. The identities of the digitized images were masked before analysis.

### Measurement of retinal thickness

Retinal thickness was measured in living mice via optical coherence tomography (OCT) at 1, 2, and 3 weeks after intravitreal injection. The mice were anesthetized via an intraperitoneal injection of 80 mg/kg ketamine and 10 mg/kg xylazine and received tropicamide eye drops as a treatment for mydriasis. Posterior ocular OCT imaging was subsequently conducted via the OCT Biµ system (Kowa). Measurements of total layer thickness were taken 200 µm from the optic nerve head manually via ImageJ software (National Institutes of Health). This experiment used 100 µM ripasudil, which significantly affected the RGC count.

### Measurement of reduced glutathione concentration

To investigate the neuroprotective effects of ripasudil, we speculated that the reduction of NMDA-induced retinal damage caused by ripasudil might involve the inhibition of oxidative stress and evaluated the concentration of reduced glutathione, which is used as an indicator of oxidative stress. The level of reduced glutathione in the retina after 1, 3, and 5 days of administration was measured by use of a GSSG/GSH quantification kit (Dojindo).

### Data analysis and statistics

All the data were expressed as means ± standard deviations (SDs). Statistical analysis was performed with the aid of JMP Pro version 11 software. All the data were the means of at least 3 independent experiments. A difference was considered significant at a probability value of < 0.05, as calculated by ANOVA and the Tukey‒Kramer test.

## Results

### RGC survival under oxidative stress

**Fig. 1:**
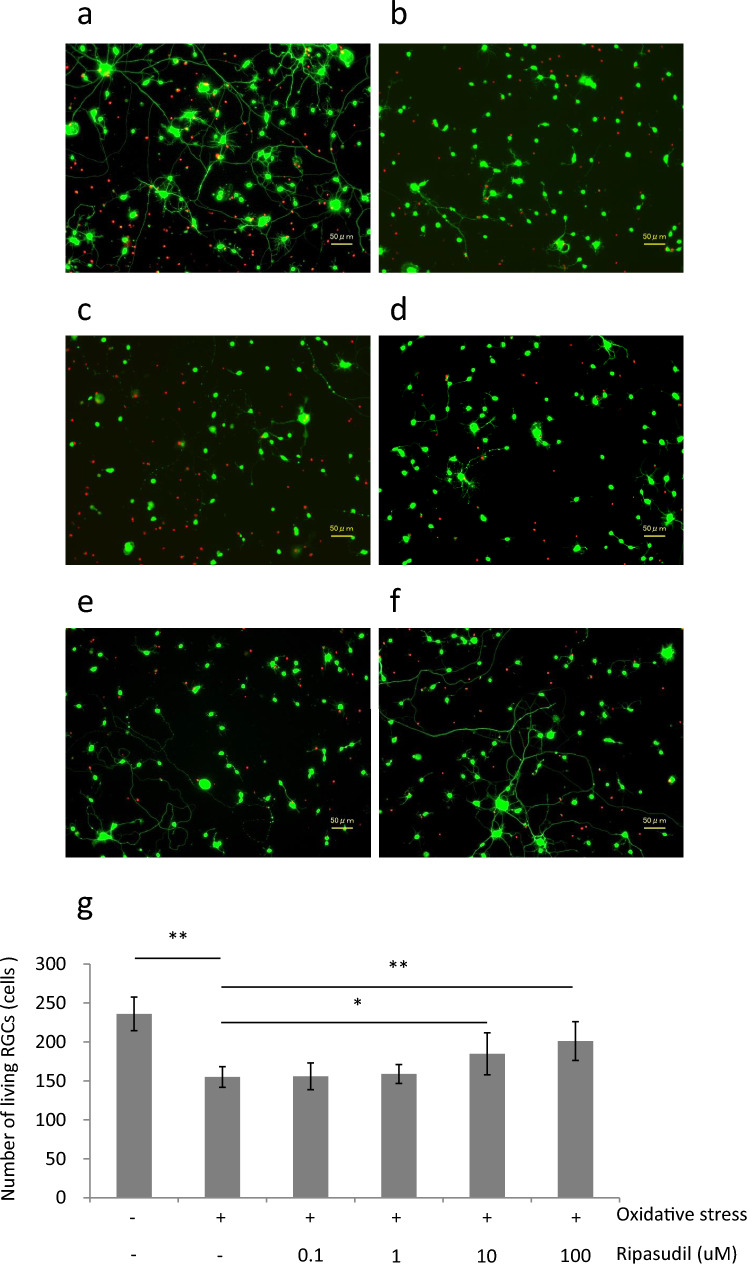
Effect of ripasudil for RGCs under oxidative stress culture. (**a**)–(**f**) are representative cell images of each group: **a** control culture (control), **b** oxidative stress (OS), **c** OS + 0.1 uM ripasudil, **d** OS + 1 uM ripasudil, **e** OS + 10 uM ripasudil, **f** OS + 100 uM ripasudil. **g** Number of living RGCs. Data are expressed as means ± SDs (n = 8). *, **P < 0.05 or 0.01 with the Tukey-Kramer test

The numbers of living RGCs in the normal culture (control), oxidative stress (OS), OS+0.1, 1, 10, and 100 µM ripasudil treatment groups were 236.0 ± 21.6, 155.0 ± 13.2, 155.9 ± 17.1, 158.9 ± 12.0, 184.8 ± 26.9, and 201.1 ± 24.8 cells, respectively. A significant difference was found between the control and OS groups (*P* < 0.01). Furthermore, significant differences were found between 10 and 100 µM ripasudil treatment and oxidative stress (*P* < 0.05 or 0.01), suggesting the significant neuroprotective effects of 10 and 100 µM ripasudil (Fig. 1).

### Effect of ripasudil on oxidative stress-induced calpain activation

**Fig. 2:**
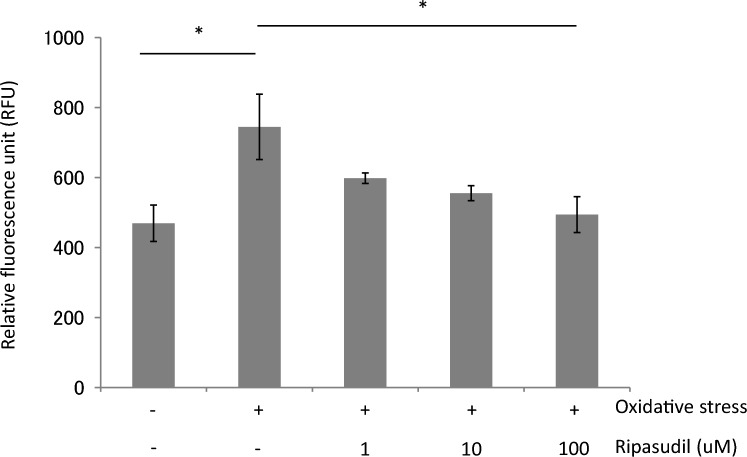
Effect of ripasudil on calpain activity under oxidative stress. Data are expressed as means ± SDs (n = 4–7). *P <0.05 with the Tukey-Kramer test

By measuring calpain activity, we showed in a previous report that necrosis was related to oxidative stress-induced RGC death [28]. To elucidate the mechanism of neuroprotection by ripasudil, we investigated calpain activity in response to ripasudil treatment under oxidative stress. The relative fluorescence units (RFUs; indices of calpain activity) of the control, OS, and 1, 10, and 100 µM ripe OS groups were 469 ± 127, 745 ± 247, 598 ± 26, 555 ± 52, and 494 ± 103, respectively. A significant difference was found between the control and OS groups (*P* < 0.05). When compared with the OS group, the 100 µM ripasudil treatment group presented significant suppression of calpain activity (*P* < 0.05) (Fig. 2).

### Effect of ripasudil on NMDA-induced retinal damage in mice

**Fig. 3:**
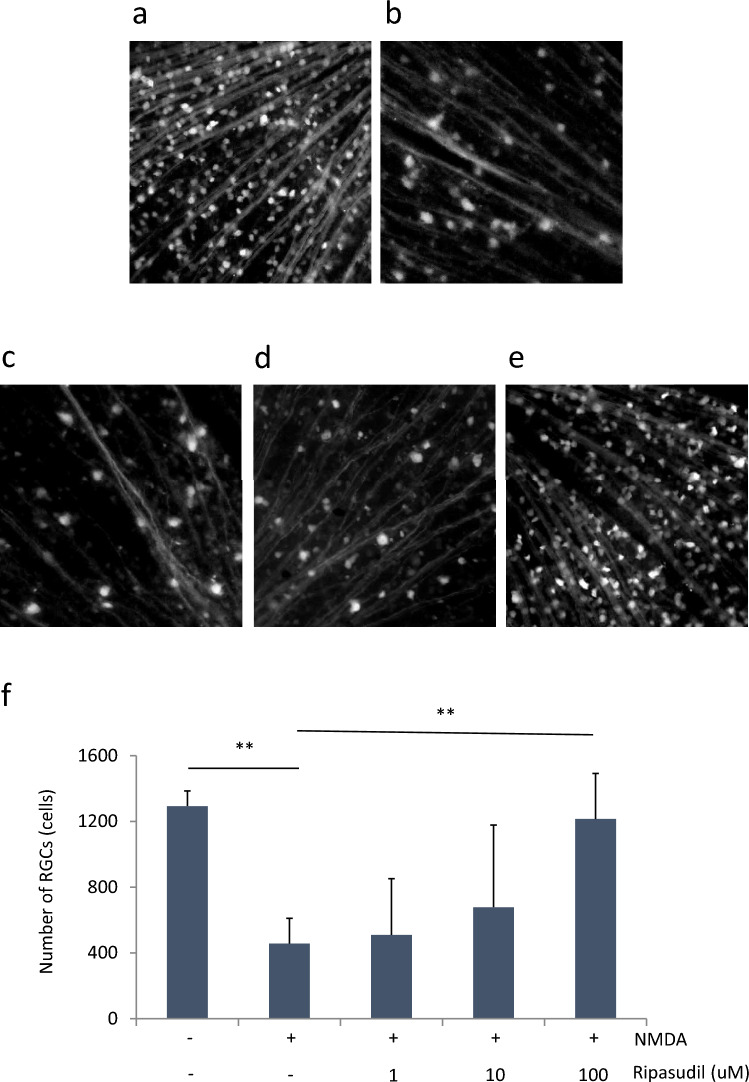
Effect of ripasudil on RGC loss in the mouse retina. RGCs were counted at 1 week after 10 mM NMDA intravitreal injection. (**a**)–(**e**) are representative retinal flat mount images of each group: **a** nontreatment (control), **b** 10 mM NMDA treatment (NMDA), **c** NMDA + 1 uM ripasudil, **d** NMDA + 10 uM ripasudil, **e** NMDA + 100 uM ripasudil. **f** Number of RGCs. Data are expressed as means ± SDs (n = 8). **P < 0.01 with the Tukey-Kramer test

In an in vitro study, we revealed that ripasudil had a neuroprotective effect on RGCs under oxidative stress. Therefore, to explore the neuroprotective effects of ripasudil in vivo, we investigated the number of RGCs in mouse whole retinal flat mounts via CFP transgenic mice. RGCs in the mouse retina were significantly damaged by 10 mM NMDA intravitreal injection (Fig. 3a, b, f). The administration of 100 µM ripasudil significantly inhibited the reduction in RGCs caused by NMDA (Fig. 3b, e, f).

**Fig. 4:**
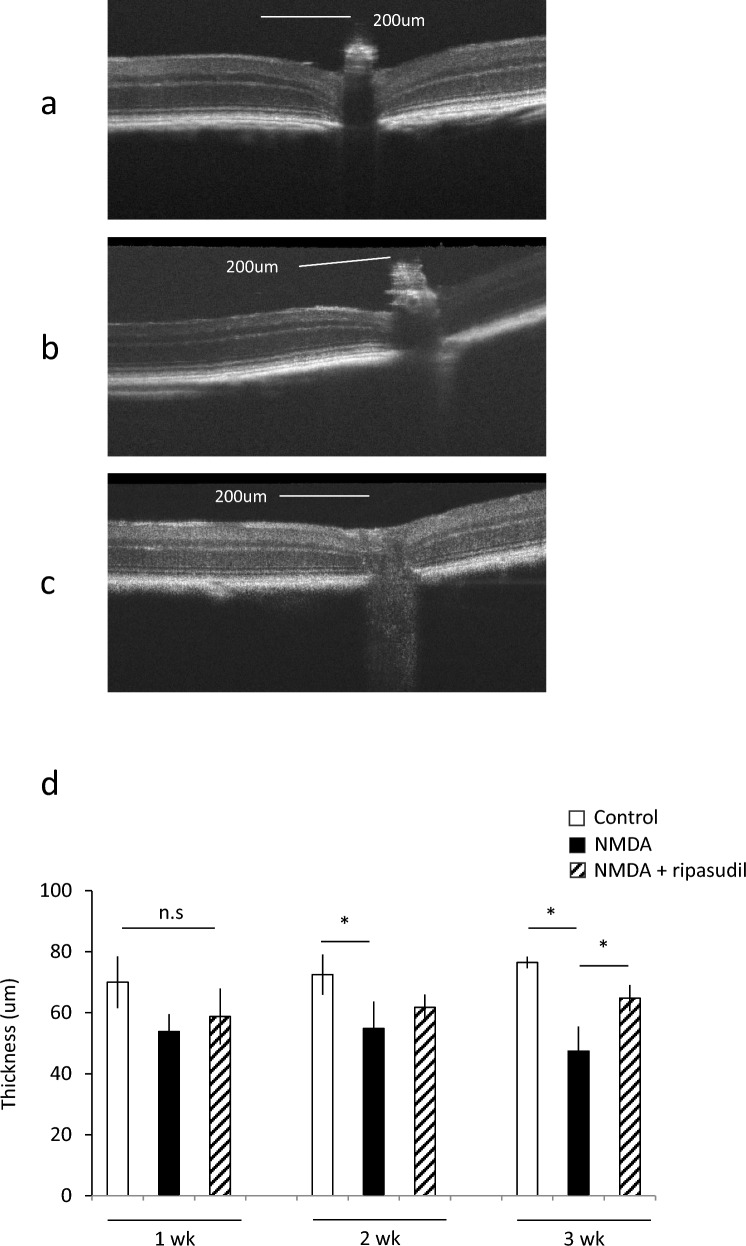
Effect of ripasudil on retinal thickness in mice. Retinal thickness was measured at 1, 2, and 3 weeks after 10 mM NMDA intravitreal injection. (**a**)–(**c**) are representative OCT images of each group: **a** nontreatment (control), **b** 10 mM NMDA treatment (NMDA), **c** NMDA + 100 uM ripasudil. **d** Retinal thickness at 1, 2, and 3 weeks after NMDA treatment. Data are expressed as means ± SDs (n = 6). *P < 0.05 with the Tukey-Kramer test

Furthermore, we investigated retinal thickness after NMDA intravitreal injection. The retinal thickness was reduced 2–3 weeks after NMDA administration (*P* < 0.05). In addition, the NMDA-induced reduction in retinal thickness was suppressed by ripasudil after 3 weeks (*P* < 0.05) (Fig. 4a, c, d). Retinal thickness after 1 week of NMDA administration tended to decrease, but the difference was not significant (*P* = 0.06) (Fig. 4d).

### Attenuation of oxidative activity by ripasudil in vivo

Because we had confirmed that ripasudil has neuroprotective effects on mouse RGCs and retinal thickness in the context of NMDA-induced retinal damage, we next aimed to clarify whether the mechanism of neuroprotection by ripasudil is related to the antioxidative effect exerted in vitro. Thus, we evaluated oxidative activity via a GSSH/GSH assay.

**Fig. 5:**
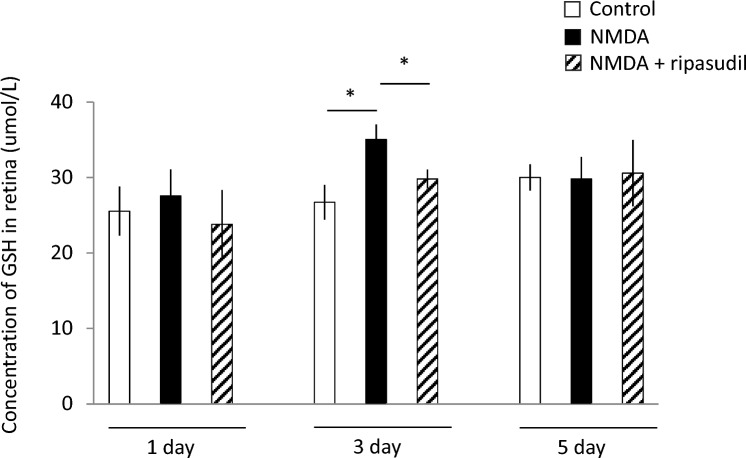
Effect of ripasudil on oxidative stress induced by NMDA injection. Oxidative stress was evaluated by use of a GSSG/GSH assay at 1, 3, and 5 days after 10 mM NMDA or simultaneous NMDA and 100 uM ripasudil intravitreal injection. Data are expressed as means ± SDs (n = 4). *P < 0.05 with the Tukey-Kramer test

Three days after NMDA administration, the glutathione level was significantly higher than that in healthy mice. Moreover, ripasudil significantly reduced the increase in oxidative activity caused by NMDA (*P* < 0.05) (Fig. 5).

## Discussion

In this study, we revealed that ripasudil has a neuroprotective effect in vivo and in vitro. Furthermore, the suppression of oxidative stress may be involved in the mechanism of neuroprotection by ripasudil. In this study, we showed that RGC viability is rescued by ripasudil at concentrations higher than 10 µM in an oxidative stress culture model (Fig. 1). In addition, calpain activity, which is related to oxidative stress-induced necrosis, is also suppressed by 100 µM ripasudil in response to oxidative stress (Fig. 2). These findings collectively indicate that ripasudil may have antioxidant properties; however, the precise mechanisms or pathways involved are unclear. A previous report indicated that oral administration of ripasudil in mice suppressed RGC death induced by nerve crush, and it was suggested that its neuroprotective effects were mediated by the *Nox* gene family, particularly *Nox1*, rather than by direct suppression of ROS production [29]. Furthermore, oxidative stress enhances the RhoA/Rho-kinase pathway [30–32], and fasudil, a rho-kinase inhibitor, reduces the various types of damage induced by oxidative stress [23, 24, 33]. Taken together, these findings suggest that the neuroprotective effects of ripasudil might be due to another mechanism of antioxidant action rather than to the direct inhibition of ROS production. In vivo, we determined that ripasudil had a neuroprotective effect via an antioxidative effect on the basis of the RGC count and retinal thickness (Figs. 3, 4 and 5). The RGC count results revealed that ripasudil inhibited the decrease in RGC count induced by NMDA injection after 1 week (Fig. 3); on the other hand, no significant difference in retinal thickness was found between the normal and NMDA-treated groups 1 week after injection (Fig. 4). It is reasonable that a decrease in the RGC count leads to a decrease in retinal thickness [34], and our data might also have been useful in this way. In addition, as demonstrated in Figure 5, an increase in glutathione levels was observed after 3 days of NMDA administration, whereas ripasudil inhibited this effect. Collectively, these results suggest that an increase in glutathione levels precedes RGC death, and consequently, thinning of the retinal layer occurs. NMDA-induced retinal damage is reportedly related to various factors, and the factors involved change and vary over time after NMDA administration [27, 35]. In particular, on the basis of previous reports, the activation of microglia is suggested to be involved in NMDA-induced delayed retinal damage [36], and rho-kinase inhibitors have shown protective effects via the inhibition of microglial activity [37, 38]. We speculate that the inhibition of microglial activity by ripasudil might also be involved in the suppression of NMDA-induced retinal damage at the late phase. However, we observed a neuroprotective effect of ripasudil in the in vitro study, where purified RGCs were cultured. This neuroprotective effect suggests that there may be additional factors, beyond glia or those unique to RGCs, involved in the mechanism of NMDA-induced injury.

Overall, our study had several limitations. First, we evaluated the neuroprotective effects of ripasudil against oxidative stress but did not further investigate other various stresses, such as hypoxia and mechanical stress, in vitro. Future studies are needed to explore the details of other stresses to clarify the mechanism of neuroprotection by ripasudil. Second, we have not yet fully elucidated the neuroprotective effects of ripasudil in vivo. Indeed, many reports have been published of rho-kinase inhibitors exhibiting protective effects owing to their antioxidant properties, as was also shown in the results of this study [23, 24, 30, 39, 40]. Moreover, rho-kinase inhibitors have been shown to have neuroprotective effects via Nox1 against oxidative stress [29]. We here only evaluated reduced glutathione, a marker of oxidative stress, in the context of NMDA-induced retinal damage. However, NMDA-induced retinal damage is not only caused by oxidative stress. Therefore, other neuroprotective mechanisms of ripasudil in addition to its antioxidant effect should also be investigated in detail in a future study. Third, we measured the whole retinal thickness, but for the early stage of retinal damage caused by NMDA injection, we evaluated the ganglion cell complex (GCC), which is defined as the 3 innermost retinal layers: the nerve fiber layer, ganglion cell layer, and inner plexiform layer. It has been reported that glaucoma likely preferentially affects these layers rather than all macular layers because they contain the axons, cell bodies, and dendrites of ganglion cells [41]. However, in the present study, we could not obtain good-quality GCC thickness data from the mouse retina because it was not possible to obtain images with high enough resolution to allow layer-by-layer analysis via the OCT Biµ system. In addition, we also considered that frozen or paraffin-embedded samples, such as HE-stained samples, were not suitable for GCC thickness measurement because of their susceptibility to artifacts. Thus, precise evaluation of the GCC is our future subject.

In conclusion, ripasudil exhibited neuroprotective effects in vivo and in vitro via antioxidative effects, which could be useful as an intraocular pressure-independent additive effect of this antiglaucoma medicine.
